# LncRNA HOTAIR promotes MPP+-induced neuronal injury in Parkinson’s disease by regulating the miR-874-5p/ATG10 axis

**DOI:** 10.17179/excli2020-2286

**Published:** 2020-08-05

**Authors:** Jingya Zhao, Hongli Li, Na Chang

**Affiliations:** 1 Department of Neurology, Huaihe Hospital of Henan University, Kaifeng 475000, Henan, China

**Keywords:** Parkinson's disease, HOTAIR, miR-874-5p, ATG10, neuronal injury

## Abstract

Parkinson's disease (PD) is a neurodegenerative disease caused by the loss of dopaminergic neurons. Long non-coding RNAs (lncRNAs) play an important role in many neurological diseases, including PD. This study aimed to investigate the role of lncRNA HOX transcript antisense RNA (HOTAIR) in PD pathogenesis and its potential mechanism. SK-N-SH cells were exposed to 1-methyl-4-phenylpyridinium (MPP^+^) to mimic PD model *in vitro.* The levels of HOTAIR, miR-874-5p and autophagy-related 10 (ATG10) were determined by quantitative real-time polymerase chain reaction (qRT-PCR) or western blot assay. Cell viability and apoptosis were assessed by Cell Counting Kit-8 (CCK-8) assay and flow cytometry. The expression of apoptosis-related proteins was measured by western blot. The levels of neuroinflammation-related factors were detected by enzyme-linked immunosorbent assay (ELISA). Commercial kits was used to monitor lactate dehydrogenase (LDH) activity, reactive oxygen (ROS) generation and superoxide dismutase (SOD) activity. The interaction among HOTAIR, miR-874-5p and ATG10 were verified by dual-luciferase reporter assay or RNA immunoprecipitation (RIP) assay. HOTAIR and ATG10 were up-regulated, and miR-874-5p was down-regulated in dose- and time-dependent manners in MPP^+^-treated SK-N-SH cells. HOTAIR knockdown reduced MPP^+^-induced neuronal damage. HOTAIR aggrandized MPP^+^-triggered neuronal injury by sponging miR-874-5p. Also, miR-874-5p attenuated MPP^+^-triggered neuronal damage by targeting ATG10. Moreover, HOTAIR regulated ATG10 expression via sponging miR-874-5p. HOTAIR promoted MPP^+^-induced neuronal injury via modulating the miR-874-5p/ATG10 axis in SK-N-SH cells.

## Introduction

Parkinson's disease (PD) is a complicated neurodegenerative disorder characterized by the absence of dopaminergic neurons in the substantia nigra (Kalia and Lang, 2015[15]). Typical symptoms of PD include rest tremor, rigidity and bradykinesia (Coelho and Ferreira, 2012[6]). Besides, 1-methyl-4-phenylpyridinium (MPP^+^) administration can mimic PD, so MPP^+^ is often used to construct PD *in vitro* models (Di Monte, 1991[8]). Therefore, alleviating MPP^+^-triggered neuronal injury is a crucial measure to ameliorate PD therapy.

A large number of studies corroborated that long non-coding RNAs (lncRNAs) implicated in biological processes such as the occurrence and development of various diseases and transcriptional regulation (Ponting et al., 2009[21]). Besides, lncRNAs exert a significant regulatory effect on central nervous system disorders, including neurodegenerative PD (Cuevas-Diaz Duran et al., 2019[7]). For instance, lncRNA SNHG1 aggravated PD progression by inducing neuroinflammation through the microRNA-7/NLRP3 axis (Cao et al., 2018[3]). LncRNA p21 expedited MPP^+^-triggered neuronal injury in SH-SY5Y cells via sponging microRNA-625 and increasing TRPM2 expression (Ding et al., 2019[9]). LncRNA MALAT1 facilitated the apoptosis of dopaminergic neuronal cells induced by MPP^+^ via regulating microRNA-205-5p/LRRK2 pathway (Chen et al., 2018[5]). Furthermore, recent studies discovered that lncRNA HOX transcript antisense RNA (HOTAIR) was prominently up-regulated in MPTP-treated PD mice and MPP^+^-stimulated SH-SY5Y cells (Liu et al., 2016[17]). Nevertheless, the specific mechanism of HOTAIR on MPP^+^-induced neuronal damage has been poorly studied.

Increasing evidence indicated that aberrantly expressed microRNAs (miRNAs) play a pivotal role in many types of neurodegenerative diseases, such as PD (Juzwik et al., 2019[14]). For example, Zhu et al. suggested that down-regulation of miR-505 relieved MPP^+^-triggered cytotoxicity in SH-SY5Y cells by inhibiting NPDC1 (Zhu et al., 2018[34]). Zhou et al. revealed that miR-128 targeted AXIN1 to modulate the apoptosis of dopamine neurons and the abundance of EAAT4 in PD (Zhou et al., 2018[33]). Wu et al. presented that miR-200a accelerated the apoptosis of striatum in PD rats via binding to DRD2 through the cAMP/PKA pathway (Wu et al., 2018[28]). Additionally, previous research exhibited that miR-874 expression was strikingly reduced in the plasma of PD patients (Chen et al., 2018[4]). However, the role of miR-874-5p in PD progression has not been investigated.

Autophagy-related 10 (ATG10) encoding an E2-like enzyme facilitates the formation of the ATG5-ATG12 complex (Hong et al., 2012[12]). The dual effects of ATG10 on HCV replication and autophagy flux are regulated by cysteine at residue 44 or 135 (Zhang et al., 2018[32]). Besides, functional polymorphism of ATG10 is closely related to breast cancer susceptibility in Chinese population (Qin et al., 2013[23]). In Parkinson's disease, ATG10 expression was remarkably elevated in MPP^+^-stimulated neuroblastoma cells (Peng et al., 2019[20]). Nevertheless, the exact mechanism of ATG10 in PD needs further investigation.

Therefore, PD models were established by treating SK-N-SH cells with MPP^+^. First, we measured the expression of HOTAIR in MPP^+^-stimulated SK-N-SH cells. What's more, the function of HOTAIR in MPP^+^-triggered neuronal damage was elucidated. Further, the potential basis of HOTAIR in PD was explored.

## Materials and Methods

### Cell culture and treatment

Human neuroblastoma cells SK-N-SH were commercially acquired from the American Type Culture Collection (ATCC; Manassas, VA, USA) and maintained in Dulbecco's modified Eagle medium (DMEM; Gibco, Carlsbad, CA, USA) with 10 % fetal bovine serum (FBS; Gibco) at 37 °C with 5 % CO_2_. Next, SK-N-SH cells were stimulated with different doses (0 mM, 0.25 mM, 0.5 mM and 1 mM) of MPP^+ ^(Sigma, St Louis, MO, USA) for 48 h or treated with 1 mM MPP^+^ for different times (0 h, 12 h, 24 h and 48 h).

### Cell transfection

Small interfering RNA (siRNA) against HOTAIR (si-HOTAIR), the siRNA control (si-NC), miR-874-5p mimics (miR-874-5p), the control mimics (miR-NC), miR-874-5p inhibitor (anti-miR-874-5p), the control inhibitor (anti-miR-NC), ATG10 overexpression vector (ATG10) and the empty overexpression vector (vector) were purchased from Ribobio (Guangzhou, China). Lipofectamine 3000 (Invitrogen, Carlsbad, CA, USA) was used to transfect oligonucleotides or plasmids into SK-N-SH cells. After stimulation with 1 mM MPP^+^ for 48 h, cell transfection was performed for 48 h.

### Quantitative real-time polymerase chain reaction (qRT-PCR)

After extracting RNA using Trizol reagent (Invitrogen), the complementary DNA (cDNA) was synthesized using PrimeScript^TM^ RT Master Mix (Takara, Dalian, China) or miRNA 1st Strand cDNA Synthesis Kit (Vazyme, Nanjing, China). Next, qRT-PCR was carried out using AceQ qPCR SYBR Green Master Mix (Vazyme) on a CFX96 PCR System (Bio-Rad, Hercules, CA, USA). Glyceraldehyde-3-phosphate dehydrogenase (GAPDH) or U6 was used as internal control. The primers were shown below: HOTAIR-F: 5'-GAGGGGAGCAGAGTTCAAGT-3', HOTAIR-R: 5'-TGGGAGGCAGCAATAGACAA-3'; miR-874-5p-F: 5'-GGCCCTGAGGAAGAACTGAG-3', miR-874-5p-R: 5'-TGAGATCCAACAGGCCTTGAC-3'; ATG10-F: 5'-AGACCATCAAAGGACTGTTCTGA-3', ATG10-R: 5'-GGGTAGATGCTCCTAGATGTGAC-3'; GAPDH-F: 5'-ACAACTTTGGTATCGTGGAAGG-3', GAPDH-R: 5'-GCCATCACGCCACAGTTTC-3'; U6-F: 5'-CTCGCTTCGGCAGCACA-3', U6-R: 5'-AACGCTTCACGAATTTGCGT-3'.

### Cell viability assay

The treated or untreated SK-N-SH cells (3×10^3^) were plated into 96-well plates. Subsequently, cells were incubated for 3 h after adding 10 μL Cell Counting Kit-8 (CCK-8) solution (Solarbio, Beijing, China). Next, Microplate Reader (BioTek, Burlington, VT, USA) was used to evaluate cell viability by measuring absorbance at 450 nm.

### Flow cytometry

SK-N-SH cells were placed into six-well plates and resuspended in PBS. Then, AnnexinV-fluorescein isothiocyanate (AnnexinV-FITC)/Propidium Iodide (PI) Apoptosis Detection kit (Invitrogen) was utilized to detect cell apoptosis. Next, the apoptosis rate was monitored using CytoFLEX flow cytometer (Beckman Coulter, Miami, FL, USA). The lower right quadrant (FITC+/PI-) represents apoptotic cells.

### Western blot assay

Cells were lysed with RIPA buffer (Solarbio). Protein samples were quantified using BCA Protein Assay Kit (Pierce, Appleton, WI, USA). Subsequently, equal amounts of protein were separated by polyacrylamide gel electrophoresis and transferred to polyvinylidene fluoride (PVDF) membranes (Millipore, Billerica, MA, USA). Then, the membranes were incubated with primary antibodies against B-cell lymphoma 2 (Bcl-2; ab196495, Abcam, Cambridge, UK), Bcl-2 associated X (Bax; ab53154, Abcam), ATG10 (ab240901, Abcam) or GAPDH (ab9485, Abcam) after blocking with 5 % skim milk. Next, the membranes were probed with the secondary antibody (ab7090, Abcam). The protein bands were visualized using Gel Doc™ XR+ System (Bio-Rad) and quantified by ImageJ software (National Institutes of Health, Bethesda, MD, USA).

### Enzyme-linked immunosorbent assay (ELISA)

SK-N-SH cells were plated in 24-well plates. Then, the levels of interleukin-6 (IL-6), interleukin 1 beta (IL-1β) and tumor necrosis factor alpha (TNF-α) in culture medium were measured using ELISA kits (R&D Systems, Minneapolis, MN, USA).

### Measurement of LDH activity 

Cytotoxicity was quantified by detecting LDH level in the culture medium using Lactate Dehydrogenase Release Assay Kit (Beyotime, Shanghai, China). In brief, after lysing SK-N-SH cells and collecting the supernatant, the reaction mixture was added to the supernatant and then incubated for 30 min at room temperature in the dark. Finally, the absorbance was monitored at 490 nm using a Microplate Reader (BioTek). LDH values were normalized by cell density.

### Detection of ROS generation

The production of ROS was detected using Reactive Oxygen Species Assay Kit (Beyotime). Briefly, the treated SK-N-SH cells were incubated with DCFH-DA for 20 min at 37 °C and then washed in serum-free medium. Subsequently, Microplate Reader (BioTek) was utilized to measure the fluorescence intensity.

### Determination of SOD activity

Cells were lysed and centrifuged at 10000×g for 15 min at 4 °C. Then, the supernatant was collected, and the activity of SOD was detected using Superoxide Dismutase Activity Assay Kit (ab65354, Abcam) for 20 min at 37 °C.

### Dual-luciferase reporter assay

The sequences of HOTAIR or ATG10 3'UTR harboring miR-874-5p wild-type or mutant binding sites were inserted into pmirGLO vector (Promega, Madison, WI, USA) to form HOTAIR WT, HOTAIR MUT, ATG10 3'UTR WT or ATG10 3'UTR MUT reporter. Next, the constructed reporter and miR-874-5p or miR-NC were co-transfected into SK-N-SH cells. The luciferase strength was analyzed using the Dual-Luciferase Reporter Assay Kit (Vazyme). 

### RNA immunoprecipitation (RIP) assay

In brief, SK-N-SH cells were lysed by RIP lysis buffer. Subsequently, cells were incubated with magnetic beads conjugated with Ago2 antibody or IgG antibody. Finally, the levels of HOTAIR and miR-874-5p were measured by qRT-PCR.

### Statistical analysis

All data were exhibited as mean ± standard deviation using Graphpad Prism 7.0 software (GraphPad, San Diego, CA, USA). Differences were analyzed using Student's t-test and one-way analysis of variance. When P <0.05, the difference was considered statistically significant.

## Results

### HOTAIR was up-regulated in MPP^+^-stimulated SK-N-SH cells

**Figure 1:**
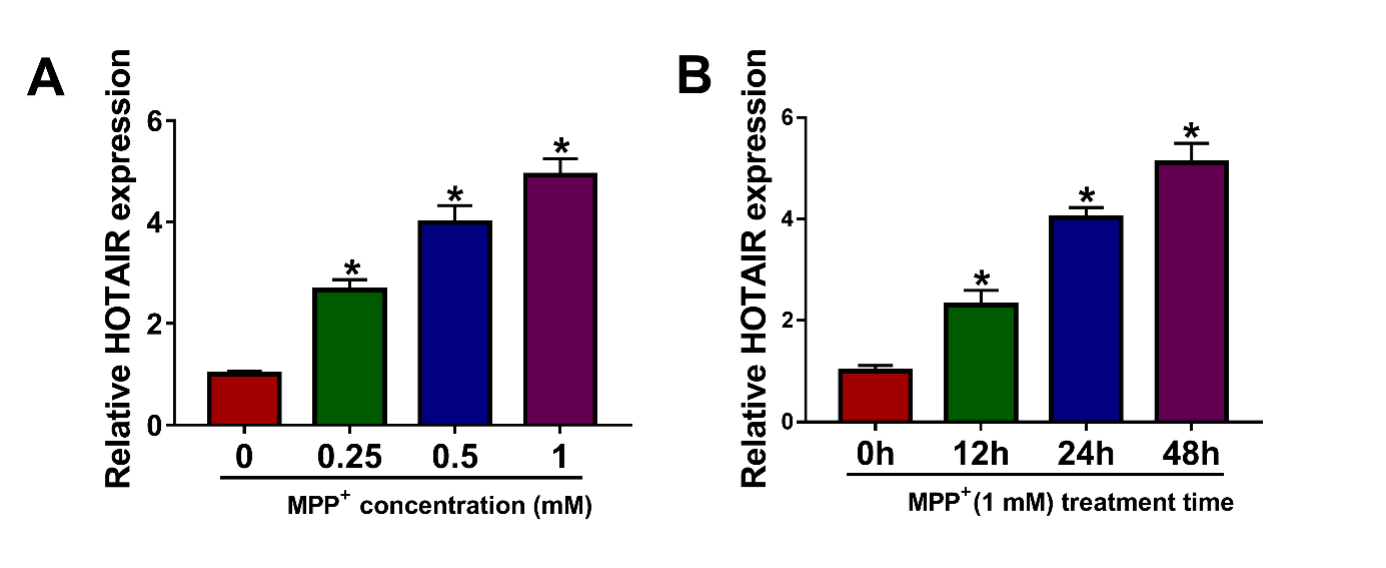
HOTAIR was up-regulated in MPP^+^-stimulated SK-N-SH cells. (A) After SK-N-SH cells were treated with different concentrations of MPP^+ ^(0 mM, 0.25 mM, 0.5 mM and 1 mM) for 24 h, the expression of HOTAIR was detected by qRT-PCR. (B) SK-N-SH cells were stimulated with 1 mM MPP^+ ^for 0 h, 12 h, 24 h or 48 h, and qRT-PCR was used to detect HOTAIR expression. *P < 0.05

In order to explore HOTAIR expression in PD cell model, SK-N-SH cells were exposed to different concentrations of MPP^+ ^(0 mM, 0.25 mM, 0.5 mM and 1 mM) for 24 h. The results of qRT-PCR exhibited that HOTAIR expression in MPP^+^-stimulated SK-N-SH cells was remarkably increased in a dose-dependent manner (Figure 1A(Fig. 1)). Besides, SK-N-SH cells were treated with 1 mM MPP^+ ^for different times (0 h, 12 h, 24 h and 48 h), and qRT-PCR analysis showed that HOTAIR level in SK-N-SH cells stimulated by MPP^+^ was markedly elevated in a time-dependent manner (Figure 1B(Fig. 1)). Therefore, 1 mM and 48 h were selected as the treatment conditions for subsequent experiments.

### Knockdown of HOTAIR reversed MPP^+^-induced neuronal injury in SK-N-SH cells

**Figure 2:**
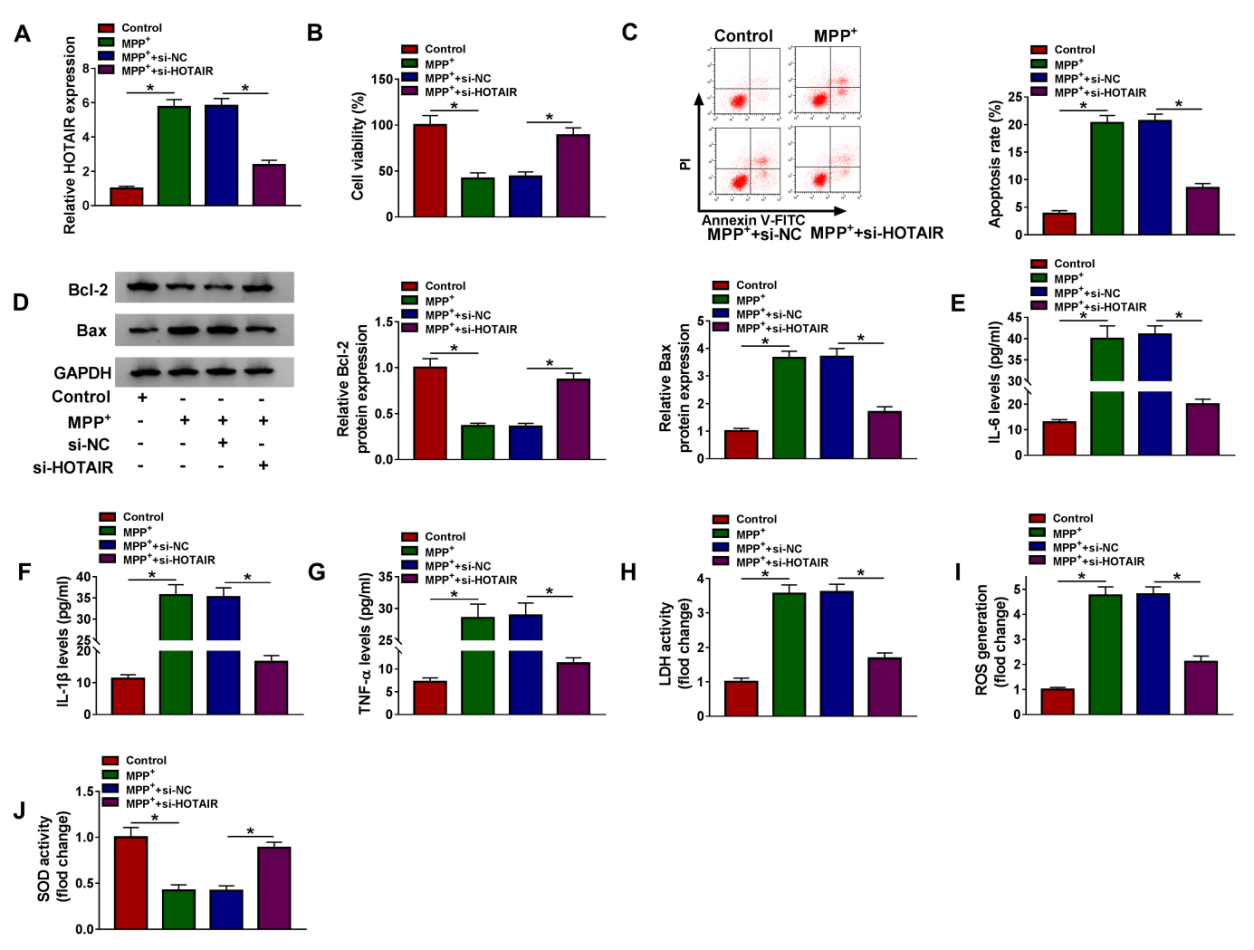
Knockdown of HOTAIR reversed MPP^+^-induced neuronal injury in SK-N-SH cells. SK-N-SH cells were introduced with si-NC or si-HOTAIR after exposure to 1 mM MPP^+ ^for 48 h. (A) The expression of HOTAIR was examined by qRT-PCR. (B) CCK-8 assay was utilized to detect cell viability. (C) Flow cytometry was used to monitor cell apoptosis. (D) The levels of apoptosis-related proteins (Bcl-2 and Bax) were measured by western blot. (E-G) The levels of IL-6, IL-1β and TNF-α were examined by ELISA. (H-J) LDH activity, ROS generation and SOD activity were measured using corresponding kits. *P < 0.05

To investigate the role of HOTAIR in PD, loss-of-function experiments were performed by transfecting si-NC or si-HOTAIR in MPP^+^-stimulated SK-N-SH cells. Firstly, qRT-PCR assay suggested that transfection with si-HOTAIR reversed the increase in HOTAIR expression induced by MPP^+ ^treatment, indicating a distinct knockdown efficiency (Figure 2A(Fig. 2)). CCK-8 analysis revealed that MPP^+ ^administration strikingly reduced the viability of SK-N-SH cells, which was reversed by HOTAIR down-regulation (Figure 2B(Fig. 2)). Flow cytometry showed that the apoptosis rate of SK-N-SH cells was overtly increased after stimulation with MPP^+^, while the effect was overturned after transfection with si-HOTAIR (Figure 2C(Fig. 2)). Consistently, MPP^+ ^stimulation resulted in a remarkable reduction of Bcl-2 expression and a noticeable increase of Bax expression, and the changes were reversed by silencing of HOTAIR (Figure 2D(Fig. 2)). Moreover, we determined the levels of IL-6, IL-1β and TNF-α using ELISA to ascertain the effect of HOTAIR on MPP^+^-triggered neuroinflammation. As displayed in Figure 2E-G(Fig. 2), silence of HOTAIR attenuated the elevation of IL-6, IL-1β and TNF-α levels caused by treatment with MPP^+^. To clarify the impact of HOTAIR on MPP^+^-induced cytotoxicity, LDH activity was detected using Lactate Dehydrogenase Release Assay Kit. The results indicated that MPP^+ ^triggered the release of LDH in SK-N-SH cells, but depletion of HOTAIR weaken the effect (Figure 2H(Fig. 2)). We also examined ROS generation and SOD activity to determine the role of HOTAIR in oxidative stress induced by MPP^+^. The results suggested that MPP^+ ^administration led to a marked increase in ROS generation and a significant decline in SOD activity, while the impacts were abrogated after introduction with si-HOTAIR (Figure 2I and 2J(Fig. 2)). Thus, these findings evidenced that HOTAIR silencing mitigated MPP^+^-induced neuronal injury in SK-N-SH cells.

### HOTAIR was a sponge of miR-874-5p

**Figure 3:**
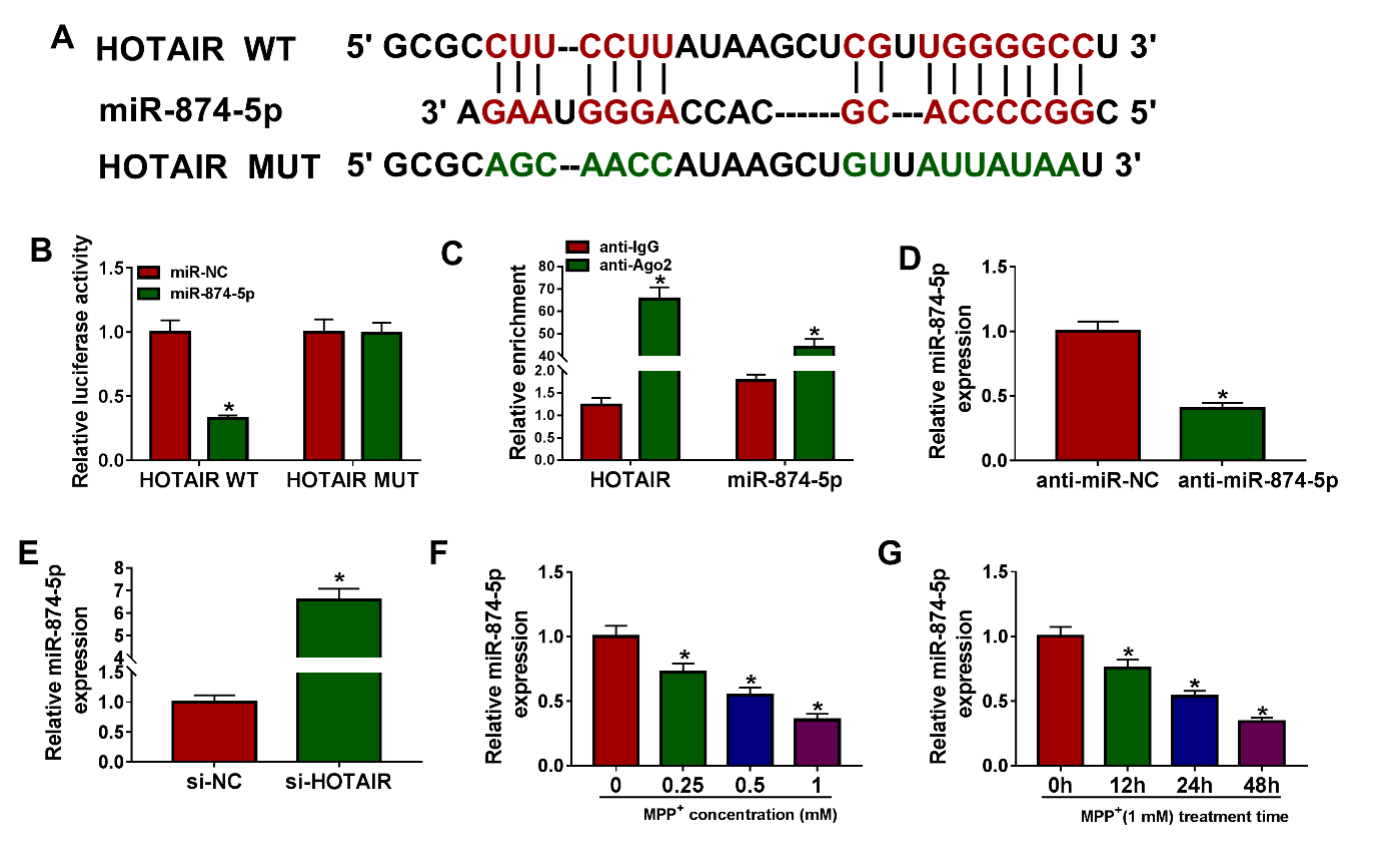
HOTAIR was a sponge of miR-874-5p. (A) LncBase Predicted v.2 predicted that HOTAIR and miR-874-5p had targeted binding sites. (B) The luciferase activity was detected using dual-luciferase reporter assay in SK-N-SH cells co-transfected with HOTAIR WT or HOTAIR MUT and miR-874-5p mimics or miR-NC. (C) RIP analysis was performed to verify the relationship between HOTAIR and miR-874-5p. (D) The inhibition efficiency of anti-miR-874-5p was determined by qRT-PCR. (E) The level of miR-874-5p was examined by qRT-PCR in SK-N-SH cells transfected with si-NC or si-HOTAIR. (F) The expression of miR-874-5p was measured in SK-N-SH cells stimulated with different doses of MPP^+ ^(0 mM, 0.25 mM, 0.5 mM and 1 mM) for 24 h. (G) The level of miR-874-5p was detected in SK-N-SH cells incubated with 1 mM MPP^+ ^for 0 h, 12 h, 24 h or 48 h. *P < 0.05.

We speculated that miR-874-5p is the target of HOTAIR using LncBase Predicted v.2 database, and the putative binding sites were shown in Figure 3A(Fig. 3). Then, dual-luciferase reporter assay was performed to validate this speculation, and the results exhibited that miR-874-5p mimics prominently reduced the luciferase activity of HOTAIR WT reporter, but did not affect HOTAIR MUT reporter (Figure 3B(Fig. 3)). In addition, RIP assay revealed that HOTAIR and miR-874-5p were evidently enriched in anti-Ago2 group relative to anti-lgG group (Figure 3C(Fig. 3)). As shown in Figure 3D(Fig. 3), miR-874-5p was significantly knocked down in the anti-miR-874-5p group compared to the control group. Moreover, the expression of miR-874-5p was detected in SK-N-SH cells introduced with si-NC or si-HOTAIR, and the qRT-PCR suggested that silencing of HOTAIR increased the level of miR-874-5p (Figure 3E(Fig. 3)). Besides, SK-N-SH cells were treated with different doses of MPP^+^ for 24 h or 1 mM MPP^+^ for varying times, and qRT-PCR analysis exhibited that miR-874-5p expression was reduced in dose- and time-dependent manners in MPP^+^-stimulated SK-N-SH cells (Figure 3F and 3G(Fig. 3)). These data indicated that miR-874-5p was a target of HOTAIR in SK-N-SH cells.

### Inhibition of miR-874-5p alleviated the inhibitory effect of HOTAIR knockdown on MPP^+^-triggered neuronal injury

**Figure 4:**
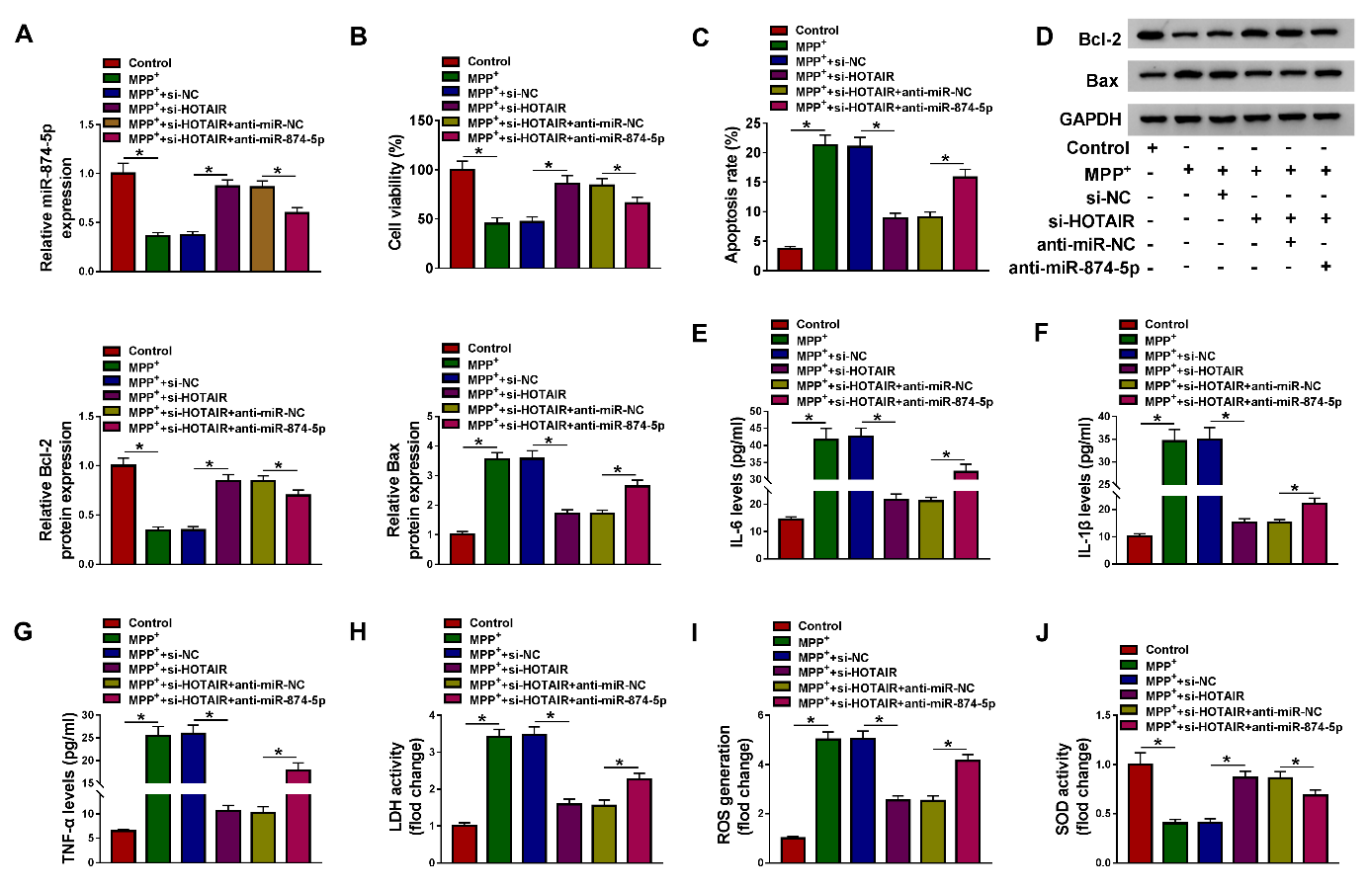
Inhibition of miR-874-5p alleviated the inhibitory effect of HOTAIR knockdown on MPP^+^-triggered neuronal injury. SK-N-SH cells were stimulated with 1 mM MPP^+ ^for 48 h and then transfected with si-NC, si-HOTAIR, si-HOTAIR+anti-miR-NC or si-HOTAIR+anti-miR-874-5p. (A) The expression of miR-874-5p was determined by qRT-PCR. (B) Cell viability was tested by CCK-8 assay. (C) The apoptosis rate was assessed by flow cytometry. (D) Western blot assay was conducted to detect the expression of Bcl-2 and Bax. (E-G) The levels of IL-6, IL-1β and TNF-α were measured using ELISA. (H-J) LDH activity, ROS generation and SOD activity were examined using commercial kits. *P < 0.05

To explore whether HOTAIR modulated MPP^+^-induced neuronal injury by targeting miR-874-5p, MPP^+^-treated SK-N-SH cells were transfected with si-NC, si-HOTAIR, si-HOTAIR+anti-miR-NC or si-HOTAIR+anti-miR-874-5p, respectively. First of all, HOTAIR knockdown elevated miR-874-5p expression, while transfection with anti-miR-874-5p alleviated the effect caused by HOTAIR depletion (Figure 4A(Fig. 4)). Besides, silencing of HOTAIR increased the viability of MPP^+^-stimulated SK-N-SH cells, which was abated through inhibition of miR-874-5p (Figure 4B(Fig. 4)). Moreover, down-regulation of HOTAIR blocked MPP^+^-induced apoptosis, including increasing Bcl-2 level and decreasing Bax level, but miR-874-5p inhibitor attenuated the inhibition of cell apoptosis induced by HOTAIR knockdown (Figure 4C and 4D(Fig. 4)). Also, transfection of anti-miR-874-5p abated the reduction of IL-6, IL-1β and TNF-α levels in MPP^+^-treated SK-N-SH cells caused by HOTAIR silencing (Figure 4E-G(Fig. 4)). Furthermore, HOTAIR depletion reduced LDH activity and ROS generation and increased SOD activity in MPP^+^-stimulated SK-N-SH cells, whereas the impacts were reversed by down-regulating miR-874-5p (Figure 4H-J(Fig. 4)). These data concluded that HOTAIR aggravated MPP^+^-triggered neuronal injury via regulating miR-874-5p.

### ATG10 was a target of miR-874-5p

**Figure 5:**
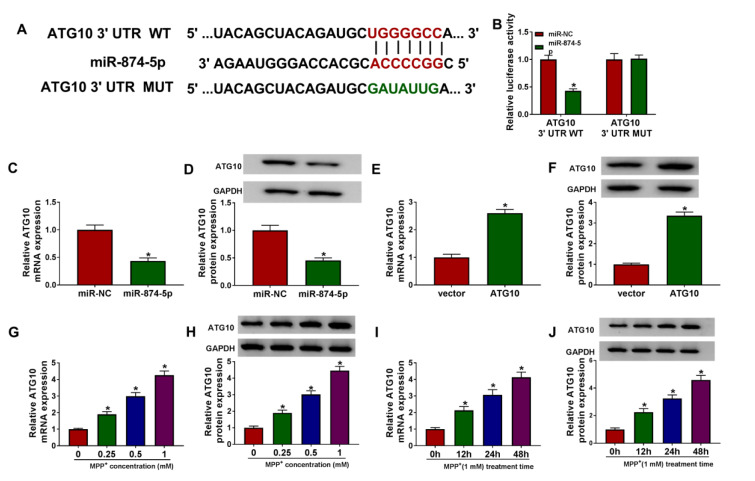
ATG10 was a target of miR-874-5p. (A) The putative binding sites between miR-874-5p and ATG10 3'UTR were displayed. (B) Dual-luciferase reporter assay was performed to verify the relationship between miR-874-5p and ATG10. (C-F) The mRNA and protein levels of ATG10 were measured in SK-N-SH cells transfected with miR-NC, miR-874-5p, vector or ATG10. (G and H) SK-N-SH cells were exposed to various concentrations of MPP^+ ^(0 mM, 0.25 mM, 0.5 mM and 1 mM) for 24 h, and ATG10 expression was examined by qRT-PCR and western blot. (I and J) SK-N-SH cells were treated with 1 mM MPP^+ ^for 0 h, 12 h, 24 h or 48 h, and ATG10 level was detected using qRT-PCR and western blot. *P < 0.05

The prediction software Targetscan showed that the 3'UTR sequence of ATG10 had putative miR-874-5p binding sites (Figure 5A(Fig. 5)). Then, dual-luciferase reporter assay was used to validate whether ATG10 was a target of miR-874-5p, and the results indicated that co-transfection of miR-874-5p and ATG10 3'UTR WT dramatically reduced the luciferase activity of SK-N-SH cells (Figure 5B(Fig. 5)). Additionally, miR-874-5p overexpression remarkably decreased the mRNA and protein levels of ATG10 in SK-N-SH cells (Figure 5C and 5D(Fig. 5)). As displayed in Figure 5E and 5F(Fig. 5), the overexpression efficiency of ATG10 was determined via qRT-PCR. Moreover, SK-N-SH cells were stimulated with varying doses of MPP^+^ for 24 h or 1 mM MPP^+^ for various times, and the results of qRT-PCR and western blot suggested that ATG10 expression was elevated in dose- and time-dependent manners in MPP^+^-treated SK-N-SH cells (Figure 5G-J(Fig. 5)). These data evidenced that ATG10 was a direct target of miR-874-5p in SK-N-SH cells.

### ATG10 attenuated the inhibitory effect of miR-874-5p on MPP^+^-induced neuronal injury

**Figure 6:**
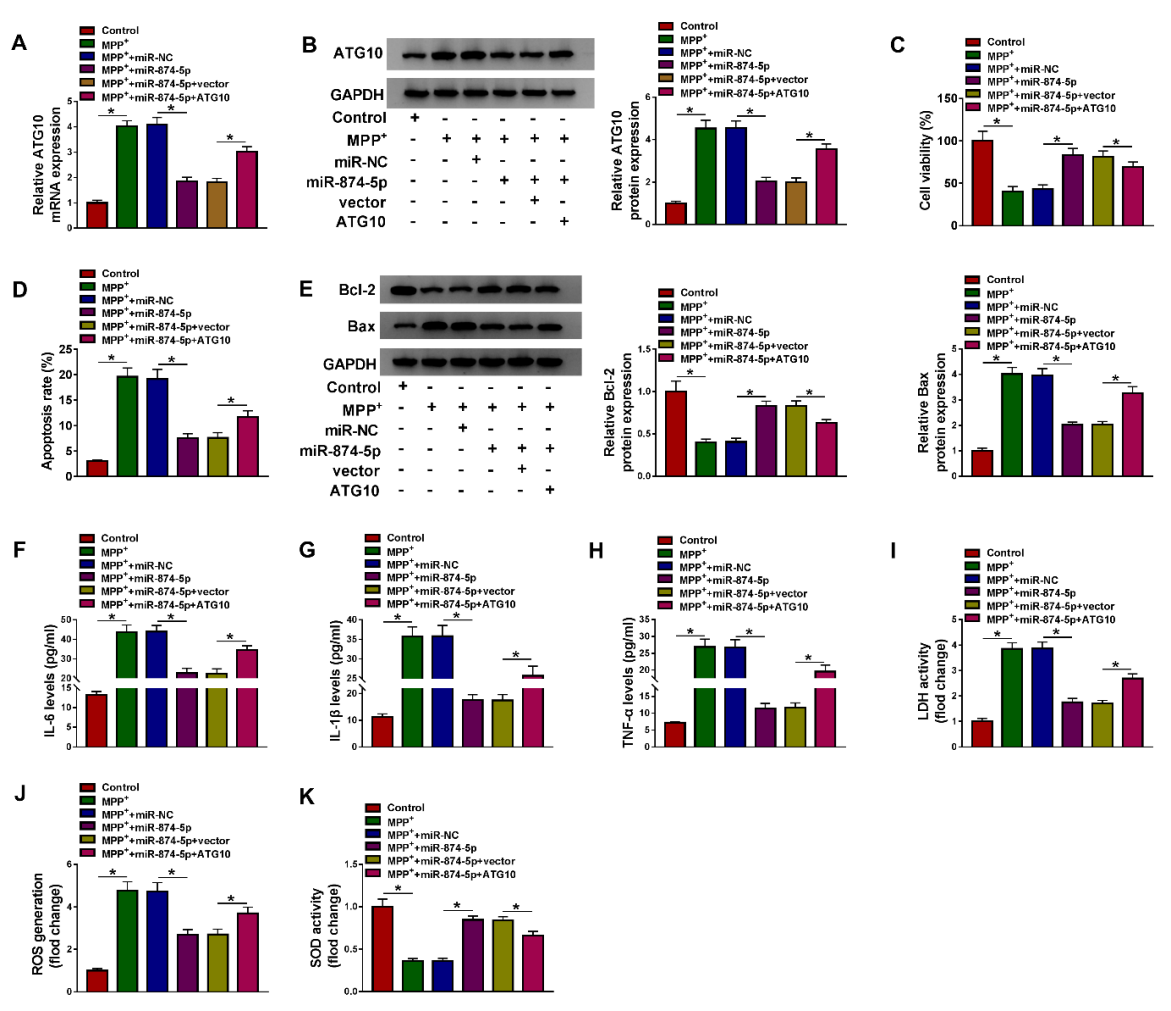
ATG10 attenuated the inhibitory effect of miR-874-5p on MPP^+^-induced neuronal injury. SK-N-SH cells were exposed to 1 mM MPP^+ ^for 48 h and then transduced with miR-NC, miR-874-5p, miR-874-5p+vector or miR-874-5p+ATG10. (A and B) The mRNA and protein levels of ATG10 were detected by qRT-PCR and western blot. (C) Cell viability was determined by CCK-8 assay. (D) Flow cytometry was used to monitor cell apoptosis. (E) The levels of apoptosis-related proteins were measured by western blot assay. (F-H) The levels of IL-6, IL-1β and TNF-α were examined using ELISA. (I-K) Commercial kits were used to monitor LDH activity, ROS generation and SOD activity. *P < 0.05

To further investigate whether miR-874-5p targeted ATG10 to mediate MPP+-induced neuronal injury, MPP^+^-stimulated SK-N-SH cells were introduced with miR-NC, miR-874-5p, miR-874-5p+vector or miR-874-5p+ATG10. The results of qRT-PCR and western blot revealed that ATG10 transfection abated the reduction in ATG10 expression level caused by miR-874-5p overexpression (Figure 6A and 6B(Fig. 6)). CCK-8 assay showed that up-regulation of miR-874-5p increased the viability of MPP^+^-treated SK-N-SH cells, which was partially reversed after transfection with ATG10 (Figure 6C(Fig. 6)). Besides, flow cytometry and western blot analysis discovered that miR-874-5p overexpression led to a distinct decrease in the apoptosis rate and Bax expression and a striking increase in Bcl-2 expression, while ATG10 overexpression diminished the inhibitory effect of miR-874-5p up-regulation on cell apoptosis (Figure 6D and 6E(Fig. 6)). Up-regulation of ATG10 overturned the decrease of IL-6, IL-1β and TNF-α levels in MPP^+^-stimulated SK-N-SH cells caused by miR-874-5p overexpression (Figure 6F-H(Fig. 6)). In addition, transfection of MPP^+^-treated SK-N-SH cells with miR-874-5p reduced LDH activity and ROS production and increased SOD activity while up-regulation of ATG10 could reverse these results (Figure 6I-K(Fig. 6)). These data manifested that miR-874-5p alleviated MPP^+^-triggered neuronal injury by targeting ATG10.

### HOTAIR regulated ATG10 expression by sponging miR-874-5p

**Figure 7:**
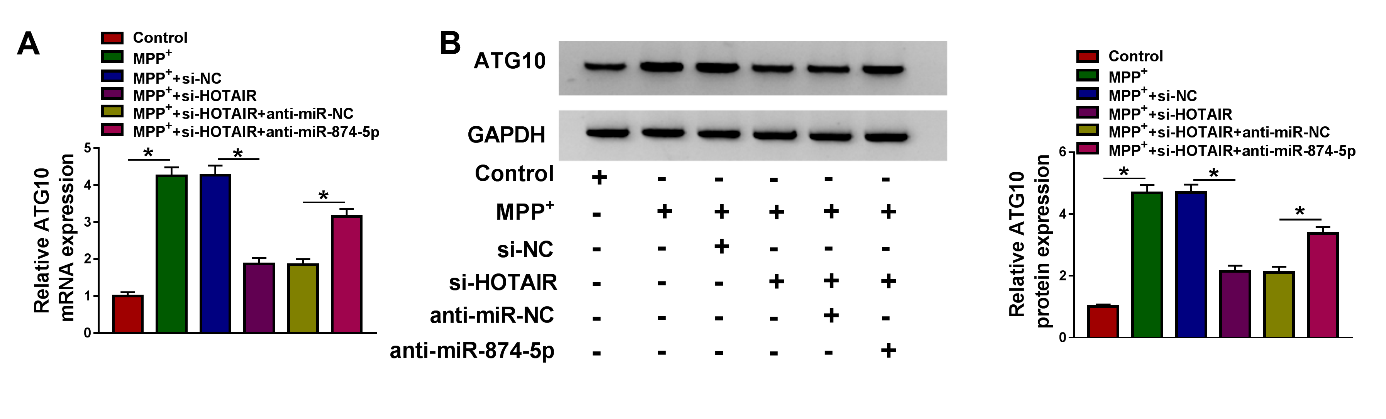
HOTAIR regulated ATG10 expression by sponging miR-874-5p. MPP^+^-treated SK-N-SH cells were introduced with si-NC, si-HOTAIR, si-HOTAIR+anti-miR-NC or si-HOTAIR+anti-miR-874-5p, respectively. (A) ATG10 mRNA expression was measured using qRT-PCR. (B) ATG10 protein expression was detected using western blot assay. *P < 0.05

To explore the association of HOTAIR and miR-874-5p in ATG10 expression, SK-N-SH cells were transfected with si-NC, si-HOTAIR, si-HOTAIR+anti-miR-NC or si-HOTAIR+anti-miR-874-5p after treatment with 1 mM MPP^+ ^for 48 h. The results of qRT-PCR and western blot revealed that HOTAIR silencing strikingly decreased the mRNA and protein levels of ATG10 in MPP^+^-treated SK-N-SH cells, and inhibition of miR-874-5p reversed the reduction of ATG10 expression caused by down-regulating HOTAIR (Figure 7A and 7B(Fig. 7)). These results indicated that HOTAIR up-regulated ATG10 expression via sponging miR-874-5p in MPP^+^-stimulated SK-N-SH cells.

For more results see the supplementary data 1 and 2.

## Discussion

One of the hallmarks of PD pathogenesis is neuroinflammation, and suppression of inflammation is a promising treatment for Parkinson's disease (Ransohoff, 2016[24]). Numerous studies have manifested that HOTAIR is a crucial regulator in inflammatory response. For example, silencing of HOTAIR decreased NF-κB-induced inflammatory factors in macrophages (Obaid et al., 2018[19]). Moreover, HOTAIR knockdown reduced LPS-mediated inflammation in hepatocytes (Zhang et al., 2020[31]). Also, HOTAIR elevated RAGE expression to expedite inflammation after acute myocardium infarction (Lu et al., 2018[18]). In the present study, depletion of HOTAIR attenuated the increase in IL-6, IL-1β and TNF-α levels caused by MPP^+^ treatment.

Besides, oxidative stress is also a hallmark of the pathogenesis of PD (Barnham et al., 2004[1]). PD induces ROS production, which mediates the death of dopaminergic neurons (Guo et al., 2018[11]; Weng et al., 2018[27]). SOD is a signature enzyme to eliminate ROS (Poprac et al., 2017[22]). We found that silence of HOTAIR alleviated MPP^+^-triggered neuronal damage by regulating apoptosis, neuroinflammation and oxidative stress. In addition, knockdown of HOTAIR attenuated MPP^+^-triggered cytotoxicity by enhancing cell viability, decreasing LDH activity and apoptosis.

Increasing evidence elucidated that lncRNAs could modulate target gene expression by serving as competing endogenous RNAs (ceRNAs) or miRNA sponges (Shi et al., 2013[25]). Numerous studies demonstrated that ceRNA mechanism exerted a significant regulatory effect on the pathogenesis of neurodegenerative disorders (Cai and Wan, 2018[2]). For example, lncRNA HOTAIR facilitated PD progression by functioning as a ceRNA for miR-126-5p to activate RAB3IP (Lin et al., 2019[16]). Moreover, bioinformatics predicted that miR-874-5p might be a target for HOTAIR. Xia et al. unveiled that miR-874-5p hindered epithelial ovarian cancer progression and potentiated paclitaxel sensitivity by targeting SIK2 (Xia et al., 2018[29]). Yao et al. disclosed that miR-874 targeted TLR4 to weaken inflammation in diabetic nephropathy (Yao et al., 2018[30]). Hence, we selected miR-874-5p as a candidate for further research and speculated that HOTAIR interacted with miR-874-5p through the ceRNA mechanism. In the current study, miR-874-5p was conspicuously down-regulated in MPP^+^-stimulated SK-N-SH cells. Further, HOTAIR reinforced MPP^+^-induced neuronal injury via sponging miR-874-5p.

Moreover, miRNAs could hamper the translation of target mRNAs via combining with the 3'UTR of target mRNAs (Eulalio et al., 2008[10]). We first confirmed that ATG10 was a target of miR-874-5p. ATG10 expression is required in autophagy initiation. Song et al. indicated that down-regulation of p62 aggravated oxidative damage in the retinal pigment epithelium by inhibiting ATG10-mediated autophagy (Song et al., 2017[26]). In colorectal cancer, high expression of ATG10 contributed to tumor metastasis (Jo et al., 2012[13]). In our research, ATG10 expression was observably increased in MPP^+^-stimulated neuroblastoma cells, which was in agreement with previous studies (Peng et al., 2019[20]). Additionally, miR-874-5p alleviated MPP^+^-induced neuronal injury via targeting ATG10. More importantly, HOTAIR modulated ATG10 expression via sponging miR-874-5p in MPP^+^-treated SK-N-SH cells, confirming the ceRNA hypothesis. 

In conclusion, HOTAIR silencing alleviated MPP^+^-triggered neuronal damage via regulating the miR-874-5p/ATG10 axis in SK-N-SH cells. These results revealed a new regulatory mechanism for HOTAIR as ceRNA and provided new biomarkers for PD treatment.

## Disclosure of interest

The authors declare that they have no conflict of interest.

## Acknowledgement

None.

## Funding

None.

## Supplementary Material

Supplementary data 1

Supplementary data 2
