# Supplementary material for: LncRNA HOTAIR promotes MPP+-induced neuronal injury in Parkinson’s disease by regulating the miR-874-5p/ATG10 axis
Source: EXCLI J. 2020 Aug 5;19:1141–53. doi: 10.17179/excli2020-2286 (PMC7527508; doi:10.17179/excli2020-2286)

EXCLI Journal 2020;19:1141-1153 – ISSN 1611-2156
Received: April 15, 2020, accepted: July 29, 2020, published: August 05, 2020

Original article:

lncRNA HOTAIR PROMOTES MPP$^+$-INDUCED NEURONAL INJURY IN PARKINSON'S DISEASE BY REGULATING THE MIR-874-5P/ATG10 AXIS

Jingya Zhao, Hongli Li, Na Chang*

Department of Neurology, Huaihe Hospital of Henan University, Kaifeng 475000, Henan, China

* **Corresponding author:** Na Chang, Department of Neurology, Huaihe Hospital of Henan University, No. 115 Ximen Street, Kaifeng 475000, Henan, China.
Tel: +86-0371-23906530. E-mail: changnachang1@163.com

http://dx.doi.org/10.17179/excli2020-2286

This is an Open Access article distributed under the terms of the Creative Commons Attribution License (http://creativecommons.org/licenses/by/4.0/).

Figure 2D

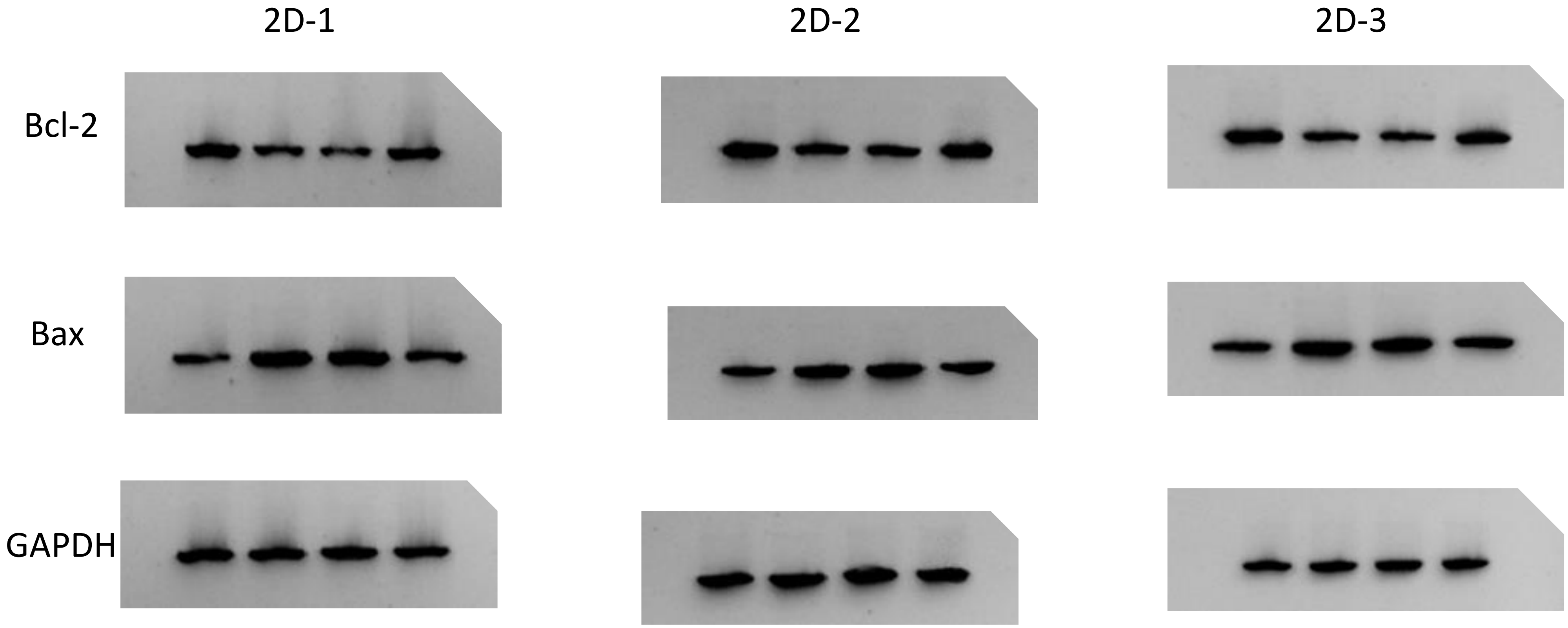

Figure 4D

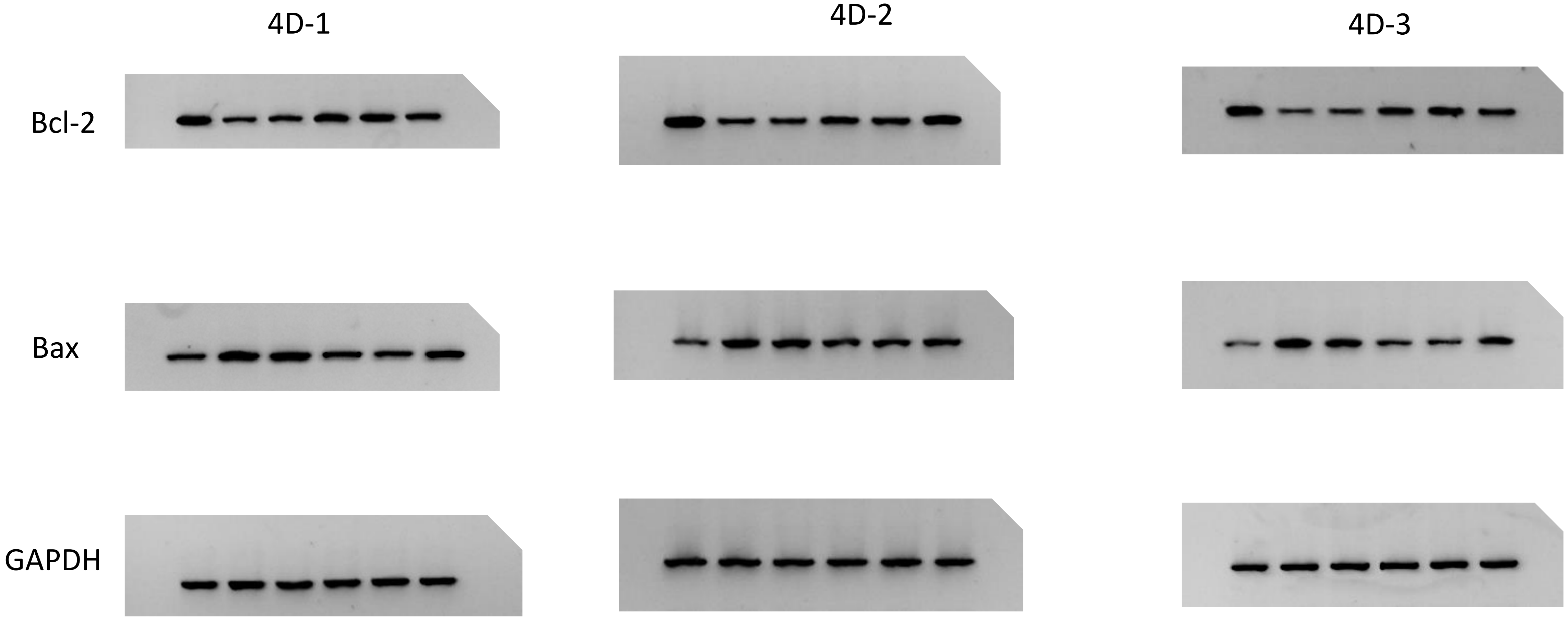

Figure 5D

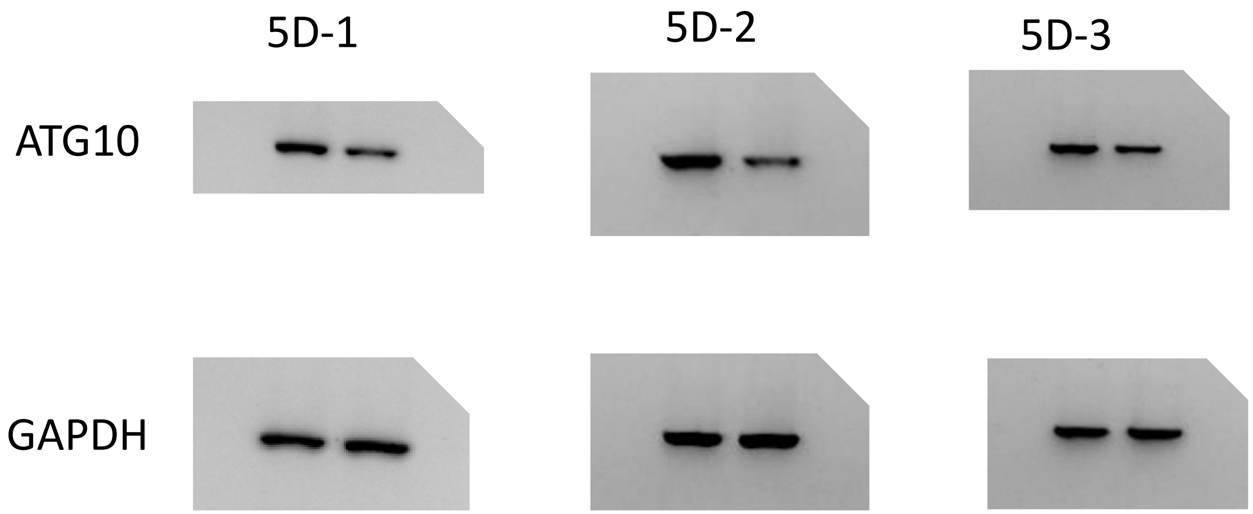

Figure 5F

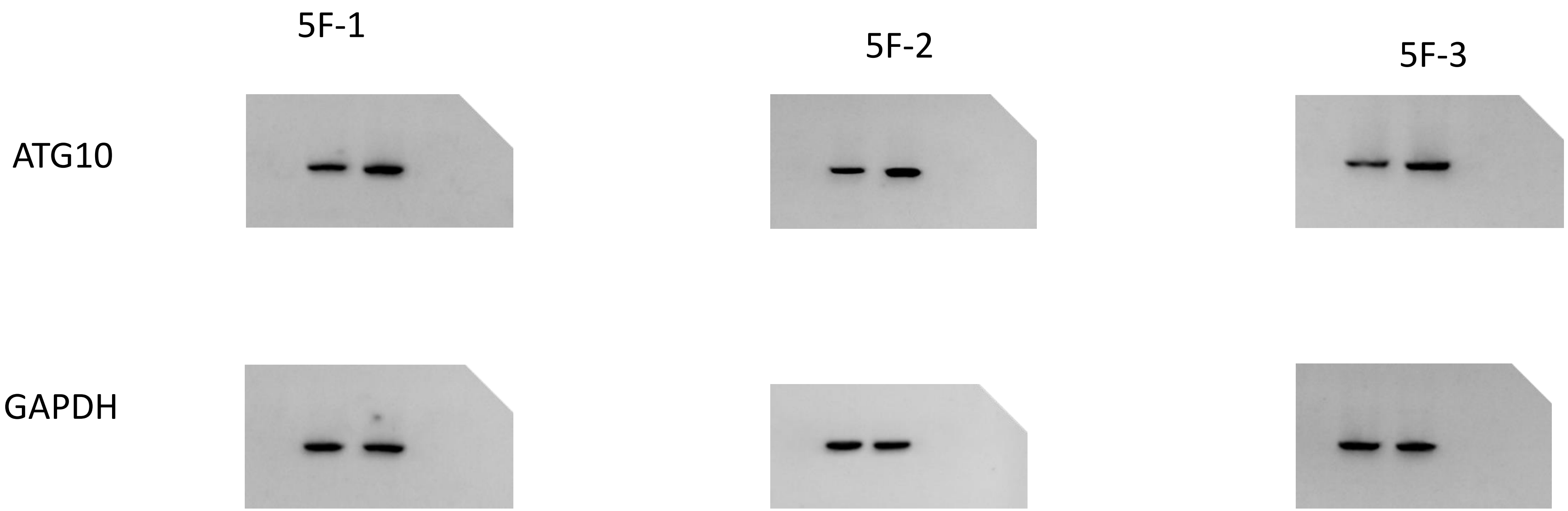

Figure 5H

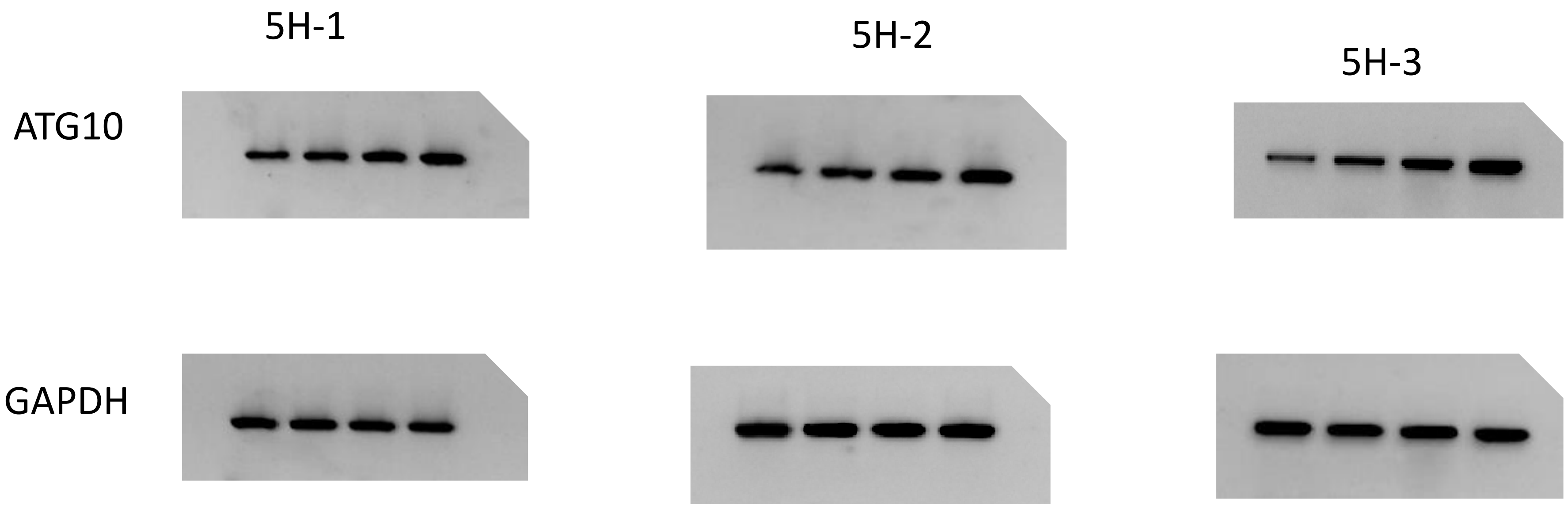

Figure 6B

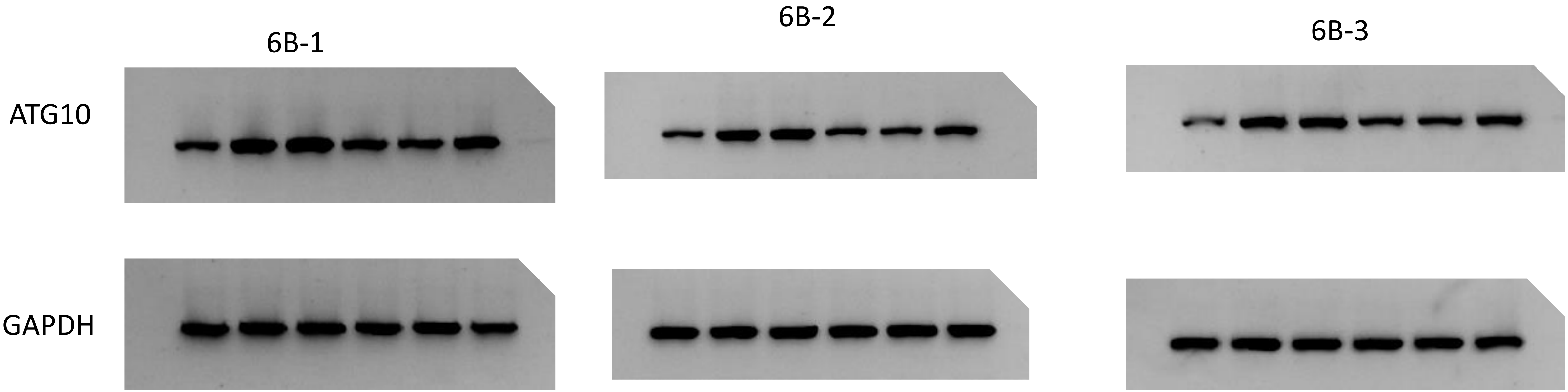

Figure 6E

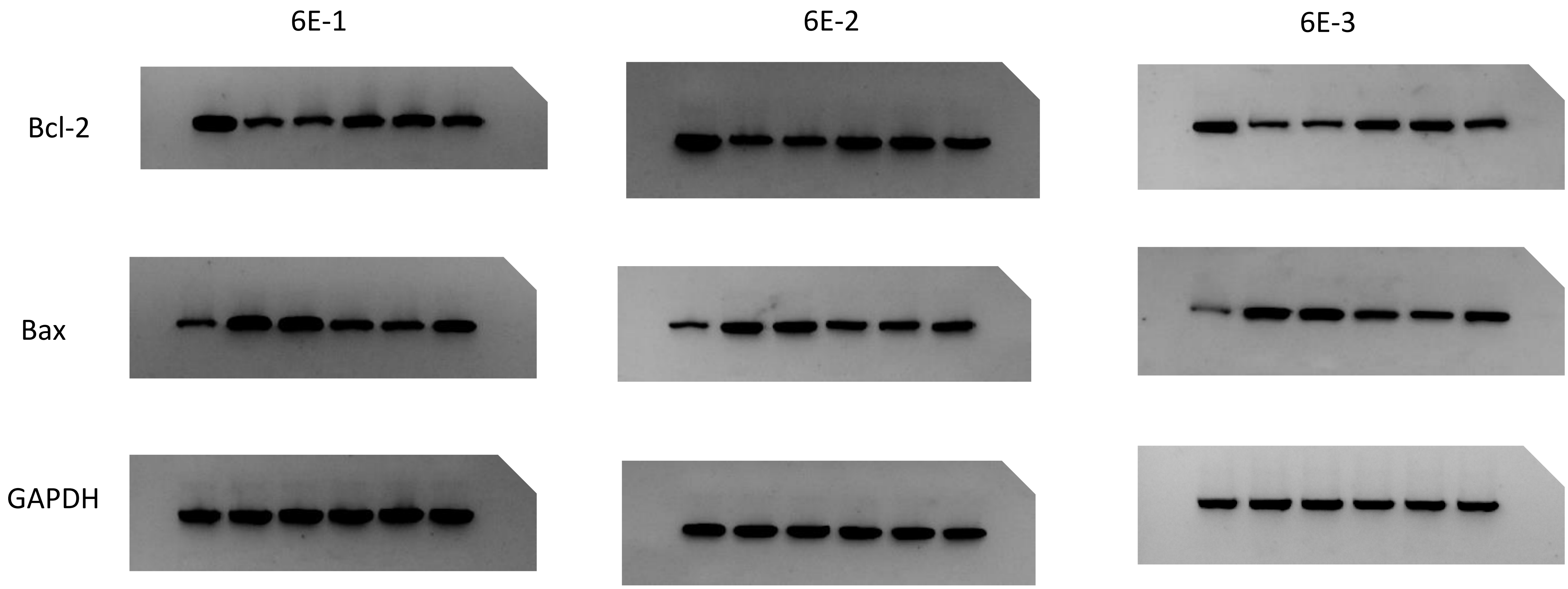

Figure 7B

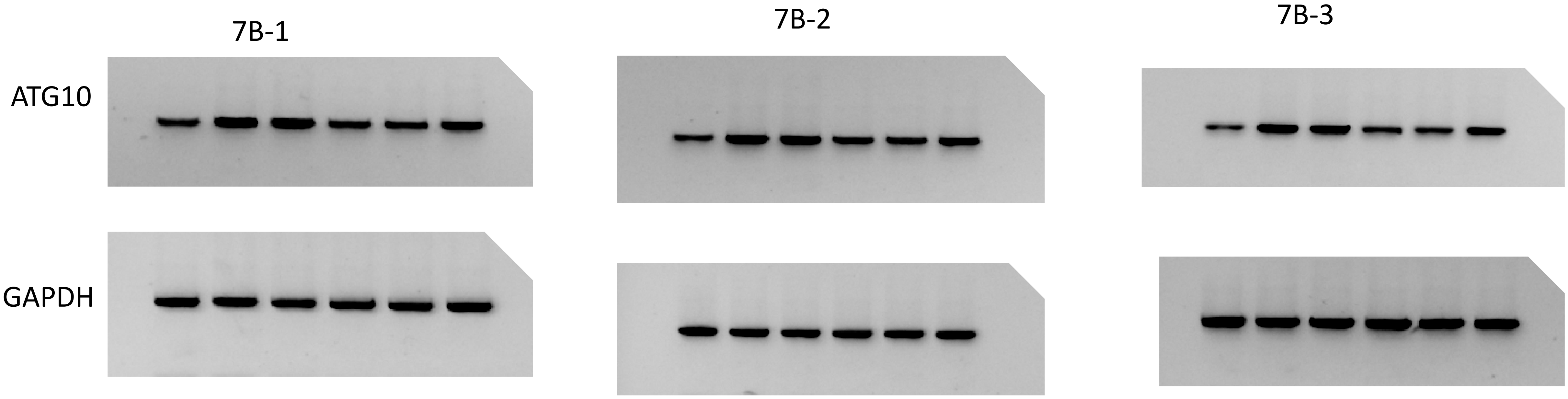

Supplement: Supplementary data 1 [file EXCLI-19-1141-s-001.pdf]
